# Rising trends and inequalities in cesarean section rates in Pakistan: Evidence from Pakistan Demographic and Health Surveys, 1990-2013

**DOI:** 10.1371/journal.pone.0186563

**Published:** 2017-10-17

**Authors:** Sarwat Mumtaz, Jinwook Bahk, Young-Ho Khang

**Affiliations:** 1 Department of Health Policy and Management, College of Medicine, Seoul National University, Seoul, South Korea; 2 Department of Public Health, Keimyung University, Daegu, South Korea; 3 Institute of Health Policy and Management, Seoul National University Medical Research Center, Seoul, South Korea; National Academy of Medical Sciences, NEPAL

## Abstract

Despite global efforts to improve maternal health, many developing countries including Pakistan have failed to achieve the target of a 75% reduction in maternal deaths by 2015. Addressing socioeconomic inequalities in access to emergency obstetric care is crucial for reducing the maternal mortality rate. This study was done to examine the time trends and socioeconomic inequalities in the utilization of cesarean section (C-section) in Pakistan during 1990–2013. We used data from the Pakistan Demographic and Health Surveys (PDHS) conducted during 1990 to 2013. All these surveys are nationally representative surveys of ever-married women aged 15–49 years with a sample size of 6611, 10,023, and 13,558 women in 1990–1991, 2006–2007, and 2012–2013, respectively, with an overall response rate of over 90%. The unit of analysis for this study was women with their most recent live birth in the five years preceding the surveys. Bivariate analyses and multivariable logistic regression models were employed to investigate the prevalence of cesarean sections according to selected sociodemographic characteristics of women. C-section rates were found to have increased during this period, with an especially significant rise from 2.7% in 1990–1991 to 15.8% in 2012–2013 with lower utilization among the non-educated women (7.5%), compared with the women with higher education (40.3%). C-section rates ranged from 5.5% in the poorest women to 35.3% in the richest women. Only 11.5% of the rural women had a C-section compared to 25.6% of the urban women. A greater likelihood of having a cesarean section was observed in the richest, highly educated, and urban-living women while there was no significant difference observed in cesarean section rates between the private and public sectors in all three surveys. To improve maternal health, routine monitoring and evaluation of the provision of emergency obstetric services are needed to address the underuse of C-section in poor and rural areas and overuse in rich and urban areas.

## Introduction

The Sustainable Development Goals have replaced the Millennium Development Goals (MDGs), with an aim of reducing the maternal mortality ratio (MMR) to less than 70 per 100,000 live births worldwide, and of ensuring healthy lives for all at all ages by 2030 [1]. Despite global efforts to improve maternal health and a 44% decline in maternal mortality worldwide, many developing countries, including Pakistan, failed to achieve the target of a 75% reduction in maternal deaths by 2015 [2]. An estimated 303,000 maternal deaths occurred in 2015, and 99% of them were reported in developing countries, particularly in Sub-Saharan Africa and South Asia [3]. This suggests an urgent need to provide equitable, high-quality, evidence-based and respectful maternity care for all women everywhere [4], specifically, universal access to emergency obstetric care should be prioritized on the global health agenda [5].

Cesarean section (C-section) is an important indicator of accessibility to the emergency obstetric care. C-section is a surgical procedure widely performed to save maternal and fetal lives and for preventing complications during labor [6]. The World Health Organization (WHO) declared that cesarean section rate should not be higher than 10%–15% [7], as unnecessary C-sections may be associated with an increased risk of maternal and neonatal mortality [8]. Simultaneously, C-sections are not always accessible to all women in many poor countries, even those in immediate need with a strong medical indication [9]. Therefore, the WHO released a new statement in 2015 regarding the rate, effective use, and indications for C-sections, and suggested that the rates should not be greater than 10% or lower than 5%, as both extremes are associated with adverse maternal and neonatal outcomes [10]. The trends in C-section rates have changed over time worldwide, with profound socioeconomic disparities across and within countries, predominantly in South Asia and Sub-Saharan Africa [11]. Cavallaro *et al*. (2013) analyzed Demographic and Health Survey (DHS) data from 26 countries in Sub-Saharan Africa and South Asia and reported C-section rates of less than 2% among the poorest groups [12]. A similar study from Mozambique revealed the underuse of C-sections among the poor and in rural areas, and overuse in advantaged groups [13]. A comparative international study using the DHS data from 45 developing countries including Pakistan found huge poor-rich inequalities in maternity care with a low coverage in rural and poor communities [14].

Pakistan is among those 10 countries of the world that are responsible for approximately 59% of the global burden of maternal deaths [3]. The basic sociodemographic and health indicators demonstrate that Pakistan is facing several demographic and health challenges, specifically in maternal and child health. Pakistan, with 192.82 million is the world sixth most populous country. The life expectancy for male and female is 65 and 67 years respectively, with an adult literacy rate of 56% “S1 Table”. Overall the MMR in Pakistan has reduced from 521 in 1990 to 178 in 2015, however, Pakistan has failed to achieve the proposed target of 130 by 2015 [15] and still the quality of obstetric and neonatal care in Pakistan is unsatisfactory [16]. In addition, the evidence clearly showed that there are significant socioeconomic disparities in maternal healthcare in Pakistan [17]. Monitoring socioeconomic inequalities plays a vital role in policy making to reduce these inequalities efficiently [18]. Specifically, exploring socioeconomic inequalities regarding access to basic emergency obstetric care is predominantly important for the improvement of maternal health in low- and middle-income countries [19]. Knowing the recent changes in rates may help in the assessment of factors regarding maternal and healthcare providers’characterstics and their effects on the rise in C-section [20]. Some studies examined association between C-section and socioeconomic status as measured by education or wealth index, using a subnational data or a single national survey data [21, 22]. However, research regarding the comparative analysis of trends and socioeconomic inequalities in utilization of C-sections has been scarce. To the best of our knowledge, no previous study has conducted a primary analysis of the rates and current status of inequalities in the C-section rate using the most recent data from Pakistan.

This study contributes to the existing literature by examining inequalities in the access and utilization of C-sections in Pakistan, using the most recent 2012–2013 Pakistan Demographic and Health Survey (PDHS) data and analyzing the sociodemographic characteristics of the study subjects, including current working status and ethnicity. This study also aimed to investigate the relationship between C-section rates and selected sociodemographic variables over time in an effort to inform policy-makers about equitable and focused strategies to end preventable maternal mortality and to improve maternal and child health in Pakistan and other South Asian countries.

## Methods

This study used globally authorized, publically available and nationally representative DHS data sets from Pakistan, conducted during 1990–2013. These data sets were downloaded from the official website of the DHS program (https://dhsprogram.com), after obtaining permission from the DHS team. The DHS is a global program supported by the United States Agency for International Development and ICF International, and conducts surveys worldwide to collect high-quality data on maternal and child healthcare, women’s empowerment, domestic violence, and knowledge of HIV/AIDS to provide guidelines in the policy-making and health evaluation programs of various nations. The same multistage stratified sampling procedure is used for all countries. Three rounds of the PDHS, conducted in 1991–1992, 2006–2007, and 2012–2013 were used for this study. All these surveys are nationally representative surveys of ever-married women aged 15–49 years. Highly trained interviewers interviewed the subjects by filling out a systematically designed questionnaire. The unit of analysis for this study was ever married women aged 15–49 years with their most recent live birth in the five years preceding the surveys. We calculated sampling weights to adjust for differences in the probability of selection and interviews among respondents. Because it was based on publicly available secondary data, this study was exempted from ethical review by the institutional review board and has no other relevant ethical considerations.

S2 Table shows the survey year, the number of total households interviewed, the sample size of the eligible ever-married women aged 15 to 49 who participated in the surveys, and the total number of women who had a live birth in the three or five years preceding the survey. In Pakistan, a total of 6611, 10,023, and 13,558 women were successfully interviewed in 1990–1991, 2006–2007, and 2012–2013, respectively, with an overall response rate of over 90%.

The outcome variable for this study was whether the last birth of a woman occurred by C-section, represented with the same questions in all surveys, such as “Was the baby delivered by cesarean section; that is did they cut your belly open to take the baby out?” or “Was the baby delivered by operation/surgery?”. This was a binary variable, with 0 meaning ‘no’ and 1 meaning ‘yes’.

The explanatory or independent variables selected for this study were included women’s age, parity; women’s education, women’s current working status, wealth index, region, area of residence (urban or rural) and ethnicity. The wealth quintiles used for this study were calculated by the DHS team by principal component analysis based on household assets, the characteristics of the home, and water and sanitation facilities, and households were categorized as belonging to the poorest, poorer, middle, richer, or the richest group [23]. We also included the place of delivery (home; public; private; and others, including non-governmental organization health arrangements) as an independent variable. The earlier rounds of PDHS lacked information about Gilgit/Baltistan because it was declared as a separate province in 2012.

We computed crude C-section rates according to women’s sociodemographic characteristics and then estimated the adjusted C-section rates standardized for maternal age and parity through a direct standardization method using the samples of the PDHS 2012–2013, as the standard population. Confidence intervals of maternal age- and parity-adjusted C-section rates were also estimated. Percentage differences in C-section rates by sociodemographic characteristics were considered as absolute magnitude of inequalities in C-section. Logistic regression was used to estimate the odd ratios of C-sections according to the women’s sociodemographic characteristics after adjustment for maternal age and parity. All analyses were performed using SAS statistical software version 9.4 (SAS Institute Inc., Cary, NC, USA).

## Results

The socio-demographic characteristics and overall cesarean section rates of women who had a live birth in five years preceding each survey are shown in Table 1. Most of the women were 25–29 years old and constituted about 29% of the total population in all three surveys. Overall, less than 10% of the women attained a higher educational level, although this trend increased remarkably from 1.2% in 1990–19991 to 9.2% in 2012–2013. With this increase in higher education, the proportion of working women also increased from 16.6% in the 1990–1991 survey to 25.0% in the 2012–2013 survey. Meanwhile, very little variation was seen in the distribution of women’s wealth index. Of the respondents, less than 20% of the women belong to the richest group in all three surveys. More than half of the women live in urban areas with the highest population in the Punjab region. The proportion of women giving birth at health facilities (public and private sectors) increased rapidly, significantly in private sector. Data from the1990-1991 survey showed that 7.3% of mothers gave birth at public hospitals and 6.3% at private hospitals. This proportion increased to 15.3% and 36.3% in the 2012–2013 survey respectively.

**Table 1:** Socio-demographic characteristics and the use of cesarean section from the Pakistan Demographic and Health Surveys (PDHS) in 1990–1991, 2006–2007, and 2012–2013.

Characteristics	PDHS 1990–1991		PDHS 2006–2007		PDHS 2012–2013	
	(*N* = 4061)		*(N* = 5677)		(*N* = 7446)	
Maternal age, (years)	Frequency	%	Frequency	%	Frequency	%
15–19	209	5.2	230	4.1	229	3.1
20–24	774	19.1	1104	19.4	1519	20.4
25–29	1202	29.6	1639	28.9	2177	29.2
30–34	859	21.2	1313	23.1	1862	25.0
35–39	600	14.8	875	15.4	1099	14.8
40–44	292	7.2	393	6.92	426	5.7
45–49	125	3.1	122	2.2	134	1.8
**Parity**						
1	634	15.6	965	17.0	1418	19.0
2–3	1234	30.4	1917	33.8	2710	36.4
4–6	1363	33.6	1837	32.4	2253	30.3
7or more	830	20.4	958	16.9	1065	14.3
**Education**						
No education	3214	79.2	3668	64.6	4155	55.8
Primary	373	9.2	854	15	1230	16.5
Secondary	427	10.5	813.4	14.3	1380	18.5
Higher	47	1.2	340.8	6	682	9.2
**Current working status**						
Working	672	16.6	1402	24.7	1857	25.0
Not working	3376	83.4	4268	75.3	5569	75.0
**Wealth index**						
Poorest	807	19.9	1289	22.7	1698	22.8
Poorer	845	20.8	1194	21.0	1544	20.7
Middle	829	20.4	1099	19.4	1464	19.7
Richer	832	20.5	1066	18.8	1469	19.7
Richest	747	18.4	1029	18.1	1272	17.1
**Urbanity**						
Urban	1184	29.2	1714	30.2	2244	30.1
Rural	2877	70.9	3962	69.8	5202	69.9
**Region**						
Punjab*	2441	60.1	3182	56.1	4180	56.1
Sindh	894	22.01	1404	24.7	1714	23.0
Khyber Pakhtunkhuwah	567	13.96	827	14.6	1117	15.0
Baluchistan	159	3.92	264	4.6	348	4.7
Gligit Baltistan	NA		NA		56	0.7
Islamabad Capital Territory (ICT)	NA		NA		31	0.4
**Ethnicity**						
Urdu	NA		375	6.6	629	8.5
Punjabi	NA		2167	38.2	2688	36.1
Sindhi	NA		660	11.6	712	9.6
Pushto	NA		864	15.2	1060	14.2
Baluchi	NA		201	3.5	352	4.7
Other	NA		1406	24.8	1904	25.6
**Place of delivery**						
Home	3488	86.4	3545	62.4	3594	48.3
Public sector	294	7.3	652	11.5	1137	15.3
Private sector	253	6.3	1448	25.5	2703	36.3
Other	4	0.1	2	0.0	4	0.0
**Cesarean section**						
No	3906	97.3	5193	91.5	6268	84.3
Yes	108	2.7	482	8.5	1171	15.8

*The Islamabad Capital Territory was included in Punjab in the PDHS 1990–1991.

Total sample size may vary because of missing values in some categories.

Cesarean section was determined for the most recent birth of women aged 15–49 years who had a live birth five years preceding the surveys.

All percentages are weighted, so the absolute number of participants does not perfectly correspond to percentages.

NA: not available.

Table 2 showed that crude C-section increased from 2.7% in 1991–1992 to 15.8% in 2012–2013, with large and increasing absolute disparities in C-section according to women’s socio-demographic characteristics. A higher education level was associated with a markedly higher C-section rate in all three surveys, specifically; in 2012–2013 this rate was upraised to 40.3%. Huge disparities in C-section rates were also found within the wealth index and region. Higher C-section rates (35.3% in 2012–2013) were seen in the richest group of women, and it was clear that the lowest rates (5.5%) were present in the poorest group. The most populated province of Pakistan, Punjab, had persistently higher C-section rate (3.0%, 10.5% and 19.1% in 1990–1991, 2006–2007 and 2012–2013 respectively) followed by Sindh (3.0%, 7.9% and 17.4% in 1990–1991, 2006–2007 and 2012–2013 respectively). Both of them showed a same pattern of increase in C-section rates during the study period as contrary to Khyber Pakhtunkhwa (KPK) and Baluchistan, which had very low rates since 1990. However, KPK improved these rates to 5.3% in 2012–2013 whereas Baluchistan still had the lowest C-section rates (1.7% in 2012–2013). In the PDHS 2012–2013, two more regions were included: Gilgit/Baltistan, and the Islamabad Capital Territory (ICT), also known as Islamabad. These two regions showed sharply contrasting rates of 3.7% in Gilgit/Baltistan and 27.7% in Islamabad. Similarly, women residing in urban areas had higher C-section rates than women in rural areas. In the most recent survey, the C-section rate was 25.6% in urban areas, as compared to 11.5% in rural areas; however, an increasing trend was also observed for rural areas. The C-section rates in the public healthcare sector were generally similar to that in private healthcare facilities. This was true for all three rounds of the PDHS. The C-section rates in the public and private sectors were 16.4% and 15.8%, respectively, in 1990–1991, 22.0% and 23.4%, respectively, in 2006–2007, and 29.0% and 31.2%, respectively, in 2012–2013. Among the different ethnic groups based on language, the Urdu-speaking population showed a higher C-section rate with an increase from 16.9% in 2006–2007 to 28.6% in 2012–2013, whereas Pushto- speaking women showed no difference during the same time period. It is also interesting to note that working and not working women showed no difference in utilizing the C-section in the earlier survey; however, this variation extended with time. The proportion of not working women had a higher C-section rate than those who work in the later surveys.

**Table 2:** Crude cesarean section rates (%) by socio-demographic characteristics from the Pakistan Demographic and Health Surveys (PDHS) in 1990–1991, 2006–2007, and 2012–2013.

Characteristics	PDHS 1990–1991			PDHS 2006–2007			PDHS 2012–2013		
	Total births	CS	%	Total births	CS	%	Total births	CS	%
**Total (N)**	4014	108	2.7	5675	482	8.5	7439	1171	15.8
**Maternal age, (years)**									
15–19	208	7	3.4	230	17	7.3	228	32	14.0
20–24	766	26	3.3	1104	107	9.7	1518	259	17.0
25–29	1186	40	3.3	1639	160	9.8	2174	353	16.3
30–34	848	26	3.1	1312	104	7.9	1861	314	16.9
35–39	592	8	1.4	874	68	7.8	1098	167	15.2
40–44	289	2	0.7	393	25	6.3	426	36	8.5
45–49	125	0	0.0	122	2	1.4	133	10	7.2
**Parity**									
1	622	37	5.9	965	155	16.1	1414	361	25.5
2–3	1226	32	2.6	1916	217	11.3	2709	532	19.6
4–6	1347	35	2.6	1836	83	4.5	2252	218	9.7
7 or more	819	5	0.6	957	27	2.9	1064	60	5.7
**Education**									
No education	3172	42	1.3	3667	161	4.4	4153	313	7.5
Primary	370	9	2.4	854	91	10.7	1227	210	17.2
Secondary	425	49	11.5	813	148	18.2	1378	374	27.1
Higher	47	9	18.6	340	82	24.1	681	274	40.3
**Current working status**									
Working	663	16	2.5	1402	80	5.7	1856	184	9.9
Not Working	3340	91	2.7	4266	402	9.4	5564	984	17.7
**Wealth index**									
Poorest	793	7	0.9	1289	29	2.3	1698	94	5.5
Poorer	840	13	1.5	1192	34	2.8	1541	108	7.0
Middle	811	5	0.6	1098	69	6.3	1464	174	11.9
Richer	826	20	2.5	1066	131	12.3	1466	346	23.6
Richest	744	63	8.5	1029	219	21.3	1271	449	35.3
**Urbanity**									
Urban	1174	75	6.4	1714	243	14.1	2243	575	25.6
Rural	2840	33	1.2	3961	240	6.1	5196	597	11.5
**Region**									
Punjab*	2424	72	3.0	3182	335	10.5	4178	798	19.1
Sindh	873	26	3.0	1404	111	7.9	1714	298	17.4
Khyber Pakhtunkhwa	564	9	1.6	825	33	3.9	1113	58	5.3
Baluchistan	153	1	0.7	264	4	1.7	348	6	1.7
Gligit Baltistan	NA						56	2	3.7
Islamabad Capital Territory							31	9	27.7
**Ethnicity**									
Urdu	NA			375	63	16.9	629	180	28.6
Punjabi	NA			2167	253	11.7	2686	603	22.5
Sindhi	NA			660	44	6.7	712	102	14.4
Pushto	NA			863	40	4.6	1057	49	4.6
Baluchi	NA			201	9	4.5	352	11	3.2
Other	NA			1405	72	5.1	1903	219	11.5
**Place of delivery**									
Home	3464	21	0.6	3545	0	0.0	3594	0	0.0
Public sector	294	48	16.4	651	143	22.0	1134	328	29.0
Private sector	252	40	15.8	1448	339	23.4	2700	843	31.2
Other	4	0	0.0	2	0	0.0	4	0	0.0

* The Islamabad Capital Territory was included in Punjab in the PDHS 1990–1991.

All percentages are weighted, so the absolute number of participants does not perfectly correspond to percentages. Cesarean section was determined for the most recent birth of women aged 15–49 years who had a live birth five years preceding the surveys.

NA: not available

As shown in Table 3, age- and parity-adjusted C-section rates increased from 3.1% in 1990–1991 to 14.9% in 2012–2013 representing almost a fivefold increase during 22 years. The greatest increase was seen in women with a higher educational level and those in the richest wealth quintile. Similar patterns of socioeconomic differences were observed in urban living women. Further, the extent of access to C-section between the province of Punjab and Baluchistan showed wide regional disparities with persistently high rates in Punjab (3.4%, 10.4% and 18.0% in 1990–1991, 2006–2007 and 2012–2013 respectively), as contrary to Baluchistan with persistently low rates (0.6%, 1.6% and 3.1% in 1990–1991, 2006–2007 and 2012–2013 respectively). However, the adjusted C-section rates in public and private health sector did not show any noticeable difference.

**Table 3:** Maternal age- and parity-adjusted cesarean section (CS) rates (%) from the Pakistan Demographic and Health Surveys (PDHS) in 1990–1991, 2006–2007, and 2012–2013.

Characteristics	PDHS 1990–1991	PDHS 2006–2007	PDHS 2012–2013
**Overall adjusted CS rates (%)**	3.1 (2.3–4.0)	8.7 (7.8–9.7)	14.9 (13.5–16.3)
**Education**			
No education	1.7 (1.0–2.4)	5.3 (4.4–6.2)	8.3 (7.0–9.7)
Primary	2.8(1.0–4.5)	10.9 (8.4–13.4)	16.9 (13.9–19.9)
Secondary	11.6(7.9–15.3)	17.2 (14.0–20.4)	25.2 (22.1–28.3)
Higher	18.5(6.3–30.7)	21.6 (16.3–27.0)	35.7 (31.1–40.3)
**Current working status**			
Working	3.1 (2.2–4.0)	9.4 (8.3–10.5)	16.3 (14.7–17.9)
Not working	3.0 (1.5–4.5)	6.8 (5.2–8.3)	10.9 (8.7–13.2)
**Wealth index**			
Poorest	1.3 (0.1–2.4)	3.5 (2.4–4.7)	7.0 (5.0–9.0)
Poorer	1.9 (0.7–3.2)	3.5 (2.3–4.7)	7.6 (5.6–9.7)
Middle	1.0 (0.3–1.7)	7.1 (5.4–8.8)	11.6 (9.1–14.2)
Richer	2.9 (1.6–4.1)	12.3 (10.0–14.5)	22.3 (19.3–25.2)
Richest	8.8 (6.6–11.0)	20.1 (17.0–23.2)	32.4 (28.3–36.5)
**Urbanity**			
Urban	6.8 (5.1–8.5)	13.6 (11.8–15.5)	23.1 (20.5–25.7)
Rural	1.5 (0.8–2.3)	6.7 (5.7–7.7)	11.5 (9.9–13.1)
**Region**			
Punjab	3.4 (2.2–4.5)	10.4 (9.0–11.8)	18.0 (15.7–20.4)
Sindh	3.5 (2.3–4.7)	8.7 (7.2–10.2)	16.4 (14.2–18.7)
Khyber pakhtunkhuwah (KPK)	2.1 (1.0–3.2)	5.0 (3.5–6.4)	4.8 (3.3–6.3)
Baluchistan	0.6 (0.0–1.3)	1.6 (0.3–2.9)	3.1 (1.6–4.5)
Gligit Baltistan	NA	NA	4.1 (2.2–6.1)
Islamabad Capital Territory (ICT)	NA	NA	23.4 (18.9–27.9)
**Ethnicity**			
Urdu	NA	16.3 (11.8–20.8)	25.5 (20.8–30.2)
Punjabi	NA	11.2 (9.5–12.9)	20.7 (17.9–23.5)
Sindhi	NA	7.7 (5.5–9.9)	14.5 (11.4–17.6)
Pushto	NA	5.6 (4.1–7.2)	4.8 (3.4–6.1)
Baluchi	NA	4.7 (0.0–9.6)	4.7 (1.5–7.9)
Other	NA	6.2 (4.6–7.8)	12.0 (9.9–14.2)
Place of delivery*			
Public sector	16.3 (11.4–21.2)	21.7 (18.0–25.3)	27.7 (24.2–31.2)
Private sector	15.8 (10.1–21.5)	22.4 (19.8–25.1)	29.5 (26.7–32.2)
Other	0.2 (-1.1–1.6)	1.8 (-2.2–5.7)	0.7(-4.3–5.7)

*Home deliveries were excluded from the analysis.

Cesarean section was determined for the most recent birth of women aged 15–49 years who had a live birth five years preceding the surveys. The study samples of PDHS 2012–2013 (*N* = 7439) was used as the standard population in the direct standardization.

NA: not available.

Age- and parity-adjusted odds ratios of C-section were estimated using logistic regression (Table 4). The odds of having a C-section increased with increasing level of education in all surveys. Similar patterns were found in wealth index. The likelihood of C-section was higher among women belonging to the richest group. In contrast, there was no significant difference in the odds of having a C-section between private and public health facilities in Pakistan.

**Table 4:** Maternal age- and parity-adjusted odd ratios of cesarean section from the Pakistan Demographic and Health Surveys (PDHS) in 1990–1991, 2006–2007, and 2012–2013.

Characteristics	PDHS 1990–1991	PDHS 2006–2007	PDHS 2012–2013
**Women’s education**			
No education	1.00 (Reference)	1.00 (Reference)	1.00 (Reference)
Primary	1.74 (0.78–3.88)	2.37 (1.67–3.37)	2.35 (1.79–3.08)
Secondary	8.55 (4.93–4.86)	3.91 (2.79–5.46)	3.70 (2.87–4.77)
Higher	14.20 (5.63–35.80)	4.66 (3.18–6.82)	5.60 (4.19–7.49)
**Working status**			
Working	1.00 (Reference)	1.00 (Reference)	1.00 (Reference)
Not working	1.05 (0.61–1.83)	1.52 (1.11–2.07)	1.65 (1.24–2.21)
**Wealth index**			
Poorest	1.00 (Reference)	1.00 (Reference)	1.00 (Reference)
Poorer	1.84 (0.44–7.71)	1.13 (0.63–2.0)	1.19 (0.75–1.90)
Middle	0.75 (0.16–3.48)	2.69 (1.60–4.52)	2.00 (1.30–3.09)
Richer	2.95 (0.79–10.98)	5.13 (3.12–8.45)	4.33 (2.94–6.38)
Richest	10.57 (3.09–36.20)	8.78 (5.30–14.53)	6.85 (4.57–10.25)
**Urbanity**			
Rural	1.00 (Reference)	1.00 (Reference)	1.00 (Reference)
Urban	6.01 (3.37–10.72)	2.29 (1.83–2.86)	2.28 (1.84–2.83)
**Region**			
Balochistan	1.00 (Reference)	1.00 (Reference)	1.00 (Reference)
Punjab	10.37 (1.99–54.09)	7.08 (3.33–15.06)	12.19 (6.13–24.21)
Sind	10.98 (2.10–57.32)	5.78 (1.22–6.35)	10.97 (5.52–21.81)
Khyber pakhtunkwa (KPK)	5.79 (1.01–33.23)	2.78 (1.22–6.35)	2.86 (1.41–5.83)
Gilgit Baltistan	NA	NA	2.14 (0.86–5.33)
Islamabad Capital Territory (ICT)	NA	NA	15.84 (7.80–32.17)
**Ethnicity**			
Baluchi	NA	1.00 (Reference)	1.00 (Reference)
Urdu	NA	4.03 (1.20–13.52)	9.08 (2.68–30.73)
Punjabi	NA	2.58 (0.76–8.79)	7.15 (2.15–23.75)
Sindhi	NA	1.76 (0.48–5.78)	4.75 (1.42–15.83)
Pushto	NA	1.12 (0.33–3.82)	1.32 (0.39–4.44)
Other		1.25 (0.37–4.26)	3.73 (1.10–12.58)
Place of delivery*			
Public sector	1.00 (Reference)	1.00 (Reference)	1.00 (Reference)
Private sector	0.99 (0.54–1.8)	1.00 (0.76–1.23)	1.08 (0.87–1.34)

*Home deliveries were excluded from the analysis.

Cesarean section was determined for the most recent birth of women aged 15–49 years who had a live birth five years preceding the surveys.

NA: not available.

## Discussion

The purpose of this study was to examine the current status and trends in socioeconomic inequalities in C-section rates in Pakistan using data from the PDHS conducted during 1990 to 2013. The findings from our analysis showed that the overall C-section rates have increased remarkably during the study period (from 2.7% in 1990 to 15.8% in 2012–2013), with huge socioeconomic disparities within the country. We observed increases in absolute magnitude (percentage difference) of inequalities in C-section rates. Our results confirmed the findings of other studies identifying wide disparities in utilization of C-section in developing countries [12, 23–26]. The multivariable logistic regression indicated that area of residence, women’s educational level and wealth index were strongly associated with having a C-section.

This rising trend in the C-section rates in Pakistan is consistent with observations from other developing countries [27–29]. Although the reasons behind this trend in developing countries are not fully understood, and are multifactorial [30], they have been thoroughly analyzed in terms of demand-side and supply-side factors in the existing literature [31]. The demand-side factors include maternal characteristics, such as high education, and the maternal choice of having a C-section to avoid labor pains, or desire for taking more bed rest which is best possible in case of a cesarean delivery, while the supply-side factors are those driven by the health care system and health professionals, such as convenient scheduling, fear of litigation and profitability for both obstetricians and institutions [31–33].

Pakistan has experienced an ongoing development in the socioeconomic and health sectors in recent decades [34]. Despite poverty, political instability, persistent income inequality, and being a victim of natural disasters and terrorism, Pakistan made substantial efforts in improving maternal and child health outcomes from 1990 to 2015 [34]. The Ministry of Health in Pakistan undertook safe motherhood initiatives as a priority in public health areas that resulted in increased skilled birth attendance, facility-based deliveries, and the provision of emergency obstetric care services in public health facilities [35]. Pakistan has a community health care workers program, ‘National Program for Family Planning and Primary Health Care’ known as the Lady Health Workers Program, since 1994; this program provides obstetric services at home at federal, provincial and district level, specifically to underserved and rural communities [36]. This program helped in provision of maternal and newborn healthcare through community health workers, made the accessibility to obstetric care possible for women’s door steps [37]. These efforts might be attributed to the overall increase in C-section, followed by the improvement in access to maternity care. However, the current wave of terrorism and deadly attacks on polio teams and lady health workers are also of great concern in terms of whether these services run smoothly [38].

It is interesting to note that in the earlier survey (PDHS1990-1991) the C-section rates were less than 2% in the poorest quintile, rural areas and women with no education, however, these rates improved in PDHS 2006–2007, after the global wave of efforts to reach the target of MDG 5 of reducing the maternal mortality rate and providing accessible emergency obstetric care to the poor and to rural areas. Still, the current PDHS survey revealed extremely large regional variations. At one extreme is the province of Baluchistan, the most underdeveloped province of Pakistan, where the C-section rates were extremely low in all three surveys which clearly showed the unavailability of life-saving obstetrical services in this region. Studies have shown that the most common reasons for the low coverage of C-sections were the unavailability of life-saving obstetrics services, insufficient provision of medicines and equipment in the available emergency obstetric health units, long distances from the basic health units, and the lack of skilled birth attendants [39, 40]. Unfortunately, all these factors may be equally responsible for this situation in Baluchistan, where long distances and mountainous landscapes without roads or proper transportation may well cause a huge geographic barrier in access to emergency obstetric care. Simultaneously, the lack of skilled birth attendants has also been reported in most urban and poor areas of Baluchistan [41]. At the other extreme is Islamabad, the capital of Pakistan, a modern and developed city in a developing country, where the C-section rate was significantly higher, at 28% in the most recent survey; this may be attributed to the more prominent socioeconomic profile of women, as well as to the easy accessibility and availability of medical facilities. This study also found noticeable ethnic inequalities in C-section rates in Pakistan. Urdu-speaking women had higher C-section rates than other women in ethnic groups, presumably because they were more likely to be educated and professional women and to live in Karachi, where emergency obstetric services are easily available. In the above context, Pakistan has dual challenges regarding C-sections rates; to monitor the overuse in advanced areas and to provide an equitable access to emergency obstetrics care to all women in all provinces.

Consistent with other prior studies [13, 42, 43], our findings showed that the women belong to the poorest households and from the rural areas had lower C-section rates than their counterparts in all study periods. The existing data have identified poverty as an important factor responsible for the low utilization of C-section in those women.

This study also highlighted education as a strong predictor of a high C-section rate, consistent with other studies in developing countries [27]. As education is directly linked with women’s autonomy, more highly educated women can make their own decision to choose to give birth through a C-section. However, high education is not always positively associated with the likelihood of having a C-section. Evidence showed a negative attitude of highly educated women towards C-section in South Korea [20], probably because education provides information on health promoting behaviour, more educated women have more knowledge about the risk of unnecessry C-section.

This study showed no significant differences in the C-section rates between public and private health facilities, as confirmed by existing data reporting similar findings [22]. This reflects the referral of complicated obstetric cases from traditional birth attendants, lady health workers, and private health facilities to public hospitals.

Although this study used high-quality, standardized, and nationally representative DHS data that facilitated comparability across populations over time, it does have some limitations. First, our analysis was restricted to the last birth, which occurred during the five years preceding the survey. Second, this study lacks information regarding the clinical indications for C-sections.

## Conclusion

The main finding of this study was an overall increasing trend and unequal coverage of C-sections in Pakistan, with lower rates among the less educated, the poorest socioeconomic stratum and rural areas, and higher rates in women with higher education, women from the richest socioeconomic stratum and from the urban areas. Further research is recommended to explore the future trends in the magnitude of these inequalities. Active and enhanced involvement of the policy sector is essential to strengthen universal health coverage and equity in maternal healthcare. To improve maternal health, routine monitoring and evaluation of the provision of emergency obstetric services are needed to address the underuse of C-section in poor and rural areas and overuse in rich and urban areas.

## Supporting information

S1 Table Selected socio-demographic and health indicators of Pakistan. (DOCX)

S2 Table Demographic profiles of the eligible* women, who participated in the surveys. (DOCX)
