# Supplementary material for: Rising trends and inequalities in cesarean section rates in Pakistan: Evidence from Pakistan Demographic and Health Surveys, 1990-2013
Source: PLoS One. 2017 Oct 17;12(10):e0186563. doi: 10.1371/journal.pone.0186563 (PMC5645133; doi:10.1371/journal.pone.0186563)
Supplement: S1 Table — (DOCX) [file pone.0186563.s001.docx]

**S1 Table. Selected socio-demographic and health indicators of Pakistan.**

| Indicators | Pakistan |
| --- | --- |
| Total population (millions)* | 192.82 |
| Female population (% of total) | 48.6 |
| Population density (people per square km of land area) | 245 |
| Population growth (annual %) | 2.0 |
| Gross domestic product per capita (current US dollars) | 1434.7 |
| Adult literacy rate (%) | 56.4 |
| Total fertility rate (births per woman) | 3.6 |
| Crude birth rate (per 1000) | 29 |
| Life expectancy, overall (years) | 66 |
| Male (years) | 65 |
| Female (years) | 67 |
| Neonatal mortality rate per 1000 live births | 46 |
| Infant mortality rate per 1000 live births | 66 |
| Under-five mortality rate per 1000 live births | 81 |
| Maternal mortality rate per 100,000 live births | 178 |

Source: The World Bank 2015 online database available from <http://data.worldbank.org/indicator>. *UNESCO Institute for Statistics 2016, available from http://data.uis.unesco.org
