# Supplementary material for: Rising trends and inequalities in cesarean section rates in Pakistan: Evidence from Pakistan Demographic and Health Surveys, 1990-2013
Source: PLoS One. 2017 Oct 17;12(10):e0186563. doi: 10.1371/journal.pone.0186563 (PMC5645133; doi:10.1371/journal.pone.0186563)
Supplement: S2 Table — (DOCX) [file pone.0186563.s002.docx]

**S2 Table. Demographic profiles of the eligible* women, who participated in the surveys.**

| Indicators | PDHS 1990-1991 | PDHS 2006-2007 | PDHS 2012-2013 |
| --- | --- | --- | --- |
| No. of households interviewed | 7193 | 95441 | 12943 |
| Sample size of eligible women | 6611 | 10023 | 13558 |
| Response rates of eligible women | 96.3 | 94.5 | 93.1 |
| No. of women who had a live birth in the last  five preceding the survey (unweighted) | 4029 | 5724 | 7461 |
| No. of women who had a live birth in the last  five years preceding the survey (weighted) | 4061 | 5677 | 7446 |

* Ever-married women aged 15-49.

Data sources: The Pakistan Demographic and Health Surveys (PDHS) 1990-1991, 2006-2007, and 2012-2013, conducted by the National Institute of Population Studies, Islamabad, Pakistan.

Abbreviations: PDHS, Pakistan Demographic and Health Survey.
